# Invariant Charge Carrier Dynamics Using a Non-Planar Non-Fullerene Acceptor across Multiple Processing Solvents

**DOI:** 10.1021/acs.jpcc.4c00708

**Published:** 2024-04-11

**Authors:** Hristo Ivov Gonev, Elena Jones, Chia-Yu Chang, Yutaka Ie, Shreyam Chatterjee, Tracey M. Clarke

**Affiliations:** † Department of Chemistry, University College London, Christopher Ingold Building, London, WC1H 0AJ, United Kingdom; ‡ The Institute of Scientific and Industrial Research (SANKEN), Osaka University, 8-1 Mihogaoka, Ibaraki, Osaka 567-0047, Japan

## Abstract

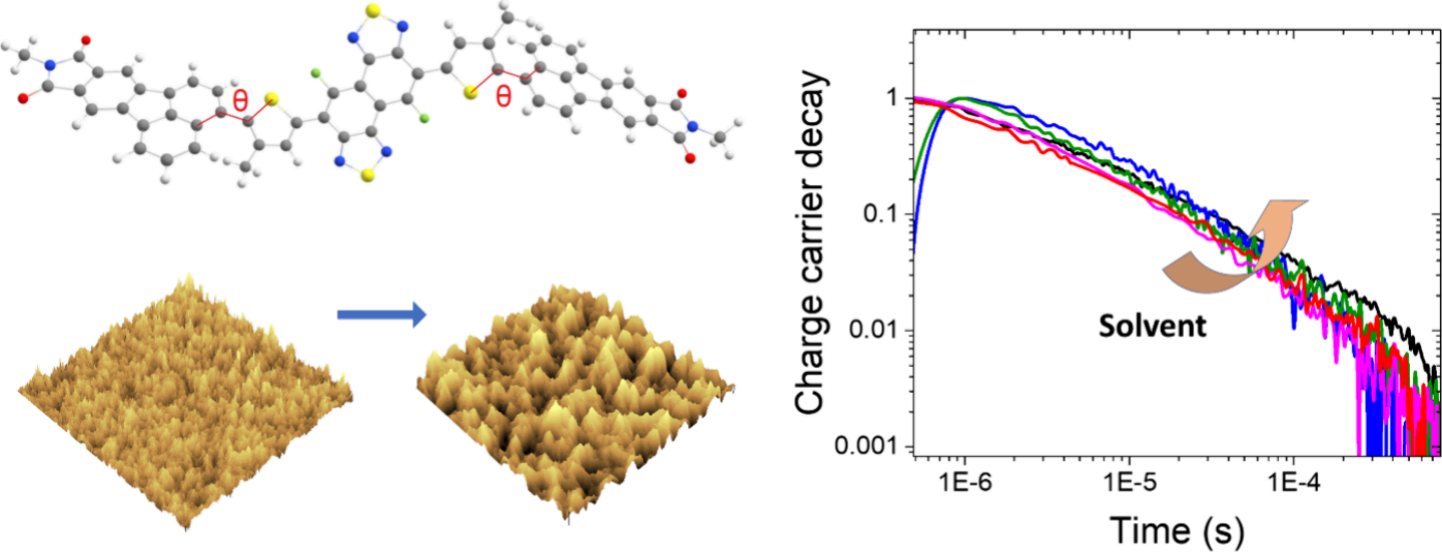

Conventional non-fullerene acceptors (NFAs) typically have planar structures that can enable improved electron mobility and produce more efficient organic photovoltaic devices. A relatively simple A-D-A′-D-A type NFA specifically designed to match with poly(3-hexylthiophene-2,5-diyl) (P3HT) for green-absorbing agrivoltaic applications has been examined using a variety of techniques: microsecond transient absorption spectroscopy, atomic force microscopy, and photoluminescence. Relatively invariant charge carrier decay dynamics are observed in the blend films across a variety of processing solvents. Raman spectroscopy in conjunction with computational studies reveals that this NFA is non-planar and that multiple conformations are present in films, while preserving the crystalline nature of P3HT. The non-planarity of the NFA therefore creates a dispersive acceptor environment, irrespective of processing solvent, and this leads to the observed relative invariance in charge carrier decay dynamics and high tolerance to morphological variation. The findings presented in this work highlight the potential of non-planar materials as acceptors in organic photovoltaic devices.

## Introduction

Organic photovoltaic (OPV) devices utilizing donor/acceptor bulk heterojunctions represent one of the most promising emerging solar cell technologies. These lightweight, thin solar cells have a multitude of advantages, including being printable from solution using cost-effective, industrial scale roll-to-roll coating techniques.^1^ They can possess short energy payback times^2^ and high red-NIR absorptivity in thin films.^3−5^ Combined, these characteristics could enable the incorporation of OPV technology into visibly transparent windows, significantly enhancing the feasibility of building-integrated photovoltaics. Non-fullerene acceptors (NFAs) have recently sparked a renaissance in the OPV field, pushing device efficiencies close to 20%.^6−9^ However, many NFAs and the conjugated polymers they are matched with are large, synthetically complex materials.^10−12^ This makes scaling up in an industrial setting difficult and means that the amount of embodied carbon in the corresponding devices is high.^13^

**Figure 1:**
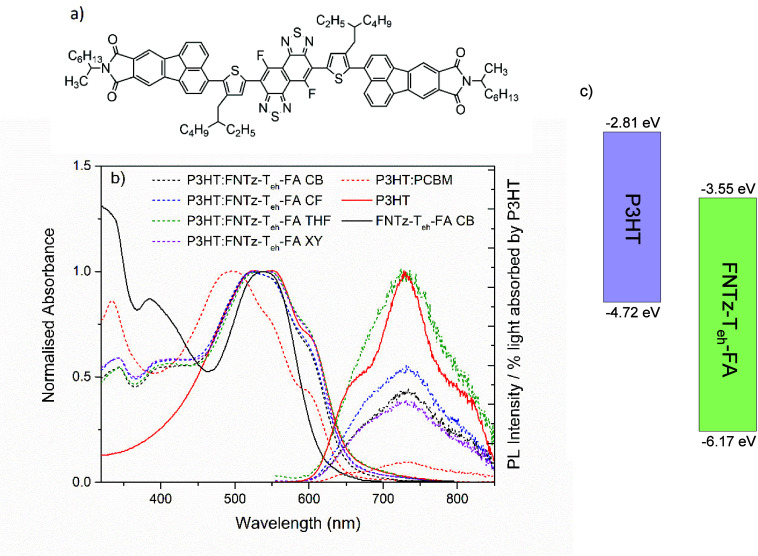
(a) The structure of the non-fullerene acceptor FNTz-T_eh_-FA and (b) the steady-state absorption and photoluminescence spectra of P3HT:FNTz-T_eh_-FA blends fabricated using different processing solvents (CB = chlorobenzene, CF = chloroform, THF = tetrahydrofuran, XY = *o*-xylene) and pristine FNTz-T_eh_-FA films fabricated using CB. The PL spectra were measured using an excitation wavelength of 540 nm and are corrected for the percentage of light absorbed at that wavelength. Also included are control samples of pristine P3HT and P3HT:PCBM films. All blend films have a donor:acceptor weight ratio of 1:1 and are annealed at 135 °C for 15 min. (c) HOMO and LUMO energy levels of P3HT and FNTz-T_eh_-FA.

In this work, we examine NFA FNTz-T_eh_-FA (3,3′-((4,9-difluoronaphtho[1,2-*c*:5,6-*c*′] bis([1,2,5]thiadiazole)-5,10-diyl)bis(3-(2-ethylhexyl)thiophene-5,2-diyl)) bis(9-(heptan-2-yl)-8*H*-acenaphtho[1,2-*f*]isoindole-8,10(9*H*)-dione)) (structure shown in Figure 1a), which has been specifically designed to energetically match with low-cost, scalable P3HT^14^ for green-absorbing agrivoltaic applications. Matching FNTz-T_eh_-FA with well-studied P3HT has an additional advantage: it will simplify the analysis of the NFA with respect to its spectroscopic properties. The synthetic complexity of FNTz-T_eh_-FA is evaluated following the method used by Po et al.^15^ (Tables S1 and S2), and this analysis shows that FNTz-T_eh_-FA possesses a relatively simple synthesis compared to other NFAs such as O-IDTBR and Y6^16^ (Figure S1).

The aim of this paper is to investigate the spectroscopic and morphological characteristics of non-fullerene acceptor FNTz-T_eh_-FA and its blends with P3HT. We are particularly interested in the effects of altering processing solvent, noting both the push toward “greener” solvents in the community but also the reported observations of substantial effects on morphology and charge carrier properties.^17,18^ We use both the conventional, chlorinated processing solvents chlorobenzene (CB) and chloroform (CF) as well as the non-halogenated tetrahydrofuran (THF) and *o*-xylene (XY), which typically show poorer yields and device performance due to reduced solubility.^19,20^ We also utilize Raman spectroscopy and computational modeling to demonstrate that FNTz-T_eh_-FA is non-planar and forms multiple conformations when blended with P3HT in a film. This non-planarity creates a dispersive environment irrespective of processing solvent and leads to high tolerance to morphological variation, enabling a relative invariance in charge carrier decay dynamics.

## Methods

### Synthesis

The synthesis of FNTz-T_eh_-FA was performed as per Chatterjee et al.^14^

### Device Fabrication

Organic solar cells were prepared with a structure of an ITO/ZnO/active layer/MoO_3_/Ag. ITO-coated glass substrates were first cleaned by ultrasonication in acetone, water, and 2-propanol for 15 min, respectively. ITO-coated glass substrates were then activated by ozone treatment for 1 h. The ZnO layer was spin-coated using the solution of zinc acetate dihydrate (99.9%, 200 mg), ethanolamine (99%, 55 μL), and 2-methoxyethanol (99.8%, 2 mL) at 3000 rpm and baked at 200 °C for 30 min in air. Subsequently, the active layer was formed by spin-coating on the ITO/ZnO electrode in a glovebox. MoO_3_ and Ag electrodes were evaporated on the top of the active layer through a shadow mask to define the active area of the devices (0.09 cm^2^) under a vacuum of 10^–5^ Pa to a thickness of 10 and 100 nm, respectively, as determined by a quartz crystal monitor. After the device was sealed from the air, the photovoltaic characteristics were measured in air under simulated AM 1.5G solar irradiation (100 mW cm^–2^) (SAN-EI ELECTRIC, XES-301S). The current density–voltage characteristics of photovoltaic devices were measured by using a KEITHLEY 2400 source meter. The EQE spectra were measured by using a Soma Optics Ltd. S-9240.

### Sample Preparation

PCBM ([6,6]-phenyl-C_61_-butyric acid methyl ester) was purchased from Solenne (>99% purity) and P3HT from Merck (*M*_w_ = 25,300). Solutions were prepared via dissolving the materials in spectroscopic grade solvents chlorobenzene (Alfa Aesar), chloroform (Thermo Fisher Scientific), tetrahydrofuran (Sigma-Aldrich), and *o*-xylene (Thermo Fisher Scientific) and stirring overnight at room temperature in a glovebox, with a N_2_ atmosphere. Concentrations used for solutions were 8 mg/mL total for P3HT:FNTz-T_eh_-FA blends at 1:1 weight ratio, 20 mg/mL for pristine FNTz-T_eh_-FA, 20 mg/mL total for P3HT:PCBM 1:1 blend ratio, and 10 mg/mL for pristine P3HT. Thin films were prepared via spin-coating for 120 s at 500 rpm from solution. Glass substrates were cleaned by separately sonicating in solutions of acetone and isopropanol for 15 min each. Blends were annealed at 135 °C for 15 min. Unless explicitly stated, all measurements were carried out under an inert atmosphere, using either a continuous nitrogen flow or an evacuated Young’s tap cuvette.

### Ground-State Absorbance

Ground-state absorbance was obtained with a Shimadzu UV-3600 i PLUS.

### Photoluminescence

Fluorescence spectra were recorded with a Horiba FluoroMax-4 spectrofluorometer and corrected for the instrument response at the exciting wavelength. Steady-state spectra were recorded at room temperature.

### Atomic Force Microscopy

The surfaces of the deposited organic films were observed by atomic force microscopy (Shimadzu, SPM9600).

### Raman Spectroscopy

Raman spectra were recorded using a custom-made backscattering setup with an Andor iDus 416 CCD camera attached to an Andor Shamrock 500i spectrograph. Raman signal was generated via excitation with a 6 ns, 10 Hz Nd:YAG laser (Spectra-Physics, INDI-40-10). The intensity of the Raman excitation was decreased with the use of neutral density filters to maintain it at 0.1 mW, measured with an ES111C sensor (Thorlabs). The excitation wavelength was selected with a versaScan L-532 OPO, and the appropriate notch filters were used in front of the spectrograph slits (100 μm).

### FT-Raman

Data was obtained on powder samples using a Bruker MultiRAM setup with an excitation wavelength of 1064 nm.

### Microsecond Transient Absorption Spectroscopy (μs-TAS)

A pump–probe micromillisecond TA spectroscopy setup was used to measure the TA spectra and kinetics. Laser pulses (repetition rate 10 Hz, pulse duration 6 ns) were generated by a Nd:YAG laser (Spectra Physics, INDI-40-10). Excitation wavelengths were selected by a versaScan L-532 OPO, and the excitation density was set in the range between 2.5 and 30 μJ cm^–2^ using neutral density filters, measured by a ES111C power meter (Thorlabs). The probe light was provided by a quartz tungsten halogen lamp (IL1, Bentham). Probe wavelength selectivity was achieved by using bandpass filters and a Cornerstone 130 monochromator (Oriel Instrument) before the detector. The TA signals were recorded with Si and InGaAs photodiodes. The signal from the photodiodes was preamplified and sent to the main amplification system with an electronic filter (Costronic Electronics), which was connected to an oscilloscope (Tektronics, DPO4034 B) and a PC.

### Computational Chemistry

Molecular structures were built in ChemDraw, with alkyl chains trimmed down to methyl groups. Calculations were performed in WebMO using the Gaussian-16 engine, B3LYP density functional method, and 6-31G(d) basis set. A vibrational frequency scaling factor of 0.96 was applied to the calculated vibrational spectra.

## Results

The steady-state absorbance spectrum of pristine FNTz-T_eh_-FA (Figure 1b) shows a maximum at 530 nm, with a 0–0 vibronic shoulder at 560 nm. These features are largely obscured when blended with P3HT and the resultant film thermally annealed. The annealed blend film shows the expected crystalline P3HT peaks at 550 and 600 nm, indicating the influence of the FNTz-T_eh_-FA present in the additional 380 nm band. The absorbance characteristics of P3HT are highly sensitive to morphology, where the intensity of the 600 nm band is a direct indicator of the extent of crystallinity in the P3HT phase.^21,22^ A comparison of the absorbance spectra of P3HT:FNTz-T_eh_-FA blends fabricated from the different solvents shows that the THF and XY blends have slightly higher intensity (by ∼10%) of the 600 nm marker band, indicating that the crystallinity of the P3HT domains in these two blends may be slightly enhanced, possibly due to the reduced solubility of P3HT in non-halogenated solvents. This observation is reproducible, noting that the 0–1 vibronic band is enhanced in the THF and XY blends as well.

Importantly, we note that the P3HT absorption in P3HT:PCBM has undergone a significant blue shift relative to that of pristine P3HT. Meanwhile, no such shift is evident in the spectra of the FNTz-T_eh_-FA blends, irrespective of processing solvent (although the strong overlap between FNTz-T_eh_-FA and P3HT means this is difficult to confirm), which indicates that PCBM reduces P3HT crystallinity much more so than FNTz-T_eh_-FA.^23^ The implication of this lack of shift is that FNTz-T_eh_-FA does not perturb the packing structure of P3HT to any significant degree and may indeed slightly enhance the crystallinity.

Although the photoluminescence spectra of the pristine FNTz-T_eh_-FA films in different solvents vary, the PL spectral shapes of all four blends are identical (Figures 1b and S2). Given the very close resemblance in spectral shape to a control P3HT:PCBM blend, the PL of the P3HT:FNTz-T_eh_-FA blends is dominated by the P3HT component. A substantial absorption overlap of P3HT and FNTz-T_eh_-FA at the excitation wavelength of 540 nm exists, and thus, both components are photoexcited. The lack of FNTz-T_eh_-FA PL in the blends therefore suggests very efficient quenching of the FNTz-T_eh_-FA excitons, either by exciton dissociation to form charge carriers (vide infra) or via singlet energy transfer to the lower energy P3HT. Furthermore, the dominant P3HT PL in the blends likely means that not all P3HT excitons are quenched via this exciton dissociation. Due to the inability to undertake selective excitation, estimation of PL quenching efficiencies relative to pristine P3HT is nontrivial. Instead, we assess the amplitudes of the PL in the blends by correcting for the percentage of light absorbed by P3HT at the excitation wavelength. We noted that a simple unweighted average of pristine P3HT and FNTz-T_eh_-FA steady-state absorption spectra was able to reproduce the relative intensity of the FNTz-T_eh_-FA absorption features at 400 nm compared to the absorption maximum (Figure S2), indicating that the P3HT is absorbing ∼50% of the total light absorbed by the blend. Using this analysis, we can see that there is still considerable PL remaining in the blends compared to pristine P3HT, particularly for the blend fabricated using THF. Given the fact that we observe decent charge carrier densities in our transient absorption spectroscopy (vide infra), this strongly suggests the presence of some singlet energy transfer in the blends. However, even if singlet energy transfer occurs, the P3HT singlets will still ultimately either relax (radiatively or non-radiatively) back to the ground state or undergo exciton dissociation.

**Figure 2:**
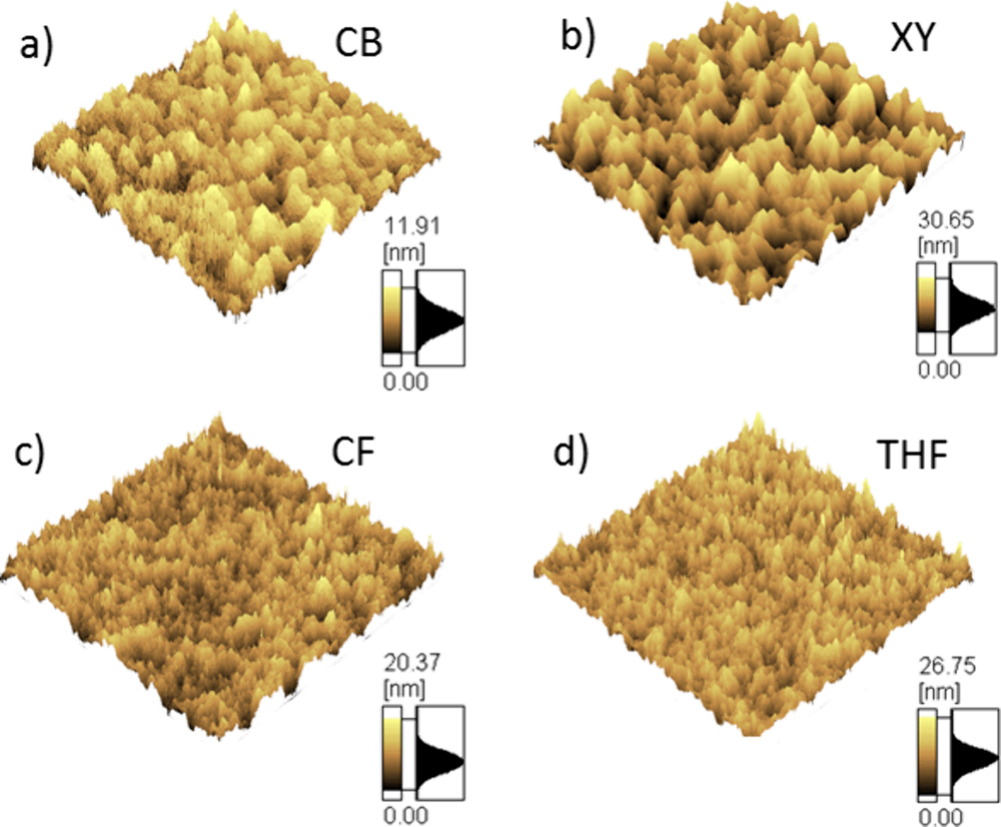
Atomic force microscopy three-dimensional surface plots for P3HT:FNTz-T_eh_-FA blend films processed using CB (a), XY (b), CF (c), and THF (d) (scale: each square shown is 2 μm × 2 μm). All blend films have a donor:acceptor weight ratio of 1:1 and are annealed at 135 °C for 15 min.

The control P3HT:PCBM blend shows a strong PL quenching of 90%. It is evident that the FNTz-T_eh_-FA blends achieve a higher intensity PL (likely caused by both singlet energy transfer and less PL quenching) compared to this PCBM control blend. The inferred reduced quenching observed for P3HT:FNTz-T_eh_-FA is consistent with that of the less perturbed P3HT observed in the ground-state absorption spectra. A greater PL intensity in a donor/acceptor blend suggests that fewer excitons have dissociated. This could occur if the donor and acceptor components are less miscible with one another, leading to a blend with a smaller interfacial area in which the domains of the individual components are more able to maintain their inherent crystallinity. In contrast, the very high level of PL quenching observed for the PCBM blend suggests a very intermixed blend with a high interfacial surface area, in which the strong intermixing disrupts the P3HT crystallinity to a large degree.

Atomic force microscopy (AFM) was applied to the P3HT:FNTz-T_eh_-FA blends fabricated from the different solvents (Figure 2 and S3). The surface morphologies of the blends using CB, CF, and THF all have similar *R*_a_ values (average of the absolute values of the surface height deviations measured from the mean plane), with *R*_a_ = 2.3 ± 0.3 nm. The low *R*_a_ values for these blends indicate relatively smooth surfaces. Only the XY blend had a larger *R*_a_ value of 4.7 nm, indicating the formation of a more segregated morphology. Estimations of the aggregate size from this data are 50 ± 10 nm for the CF blend, ∼80 ± 10 nm for the THF blend, ∼130 ± 20 nm for CB, and ∼290 ± 30 nm for the XY blend. Interestingly, there is a correlation between the aggregate size and the corrected PL amplitude. The two films with the largest average aggregate sizes (XY and CB) show the least PL, while the smaller aggregate blends show the greatest PL (THF and CF). This is inconsistent with standard interpretations of PL quenching but is consistent with the singlet energy transfer hypothesis, noting that energy transfer is likely to be more prevalent in a miscible, intermixed blend.

**Figure 3:**
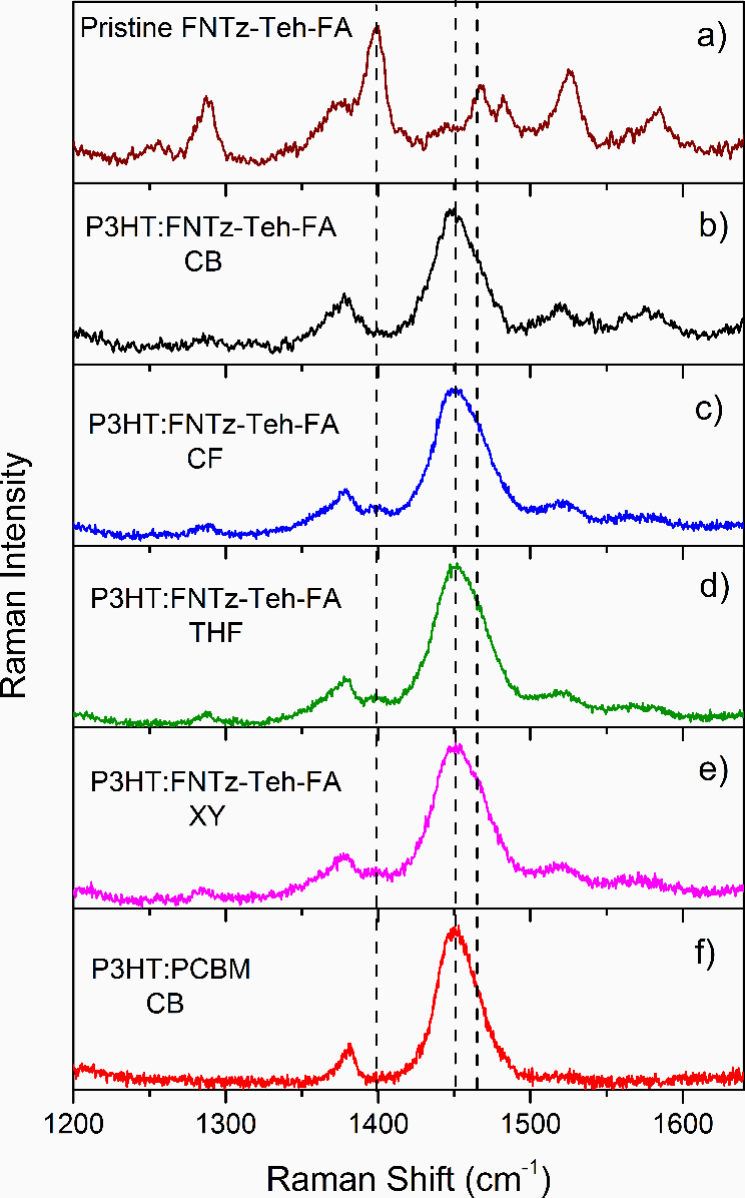
Raman spectra measured for a pristine FNTz-T_eh_-FA film (a), P3HT:FNTz-T_eh_-FA blend films fabricated using different processing solvents (b–e), and a control P3HT:PCBM film (f) using an excitation wavelength of 488 nm. All blend films have a weight ratio of 1:1 and are annealed at 135 °C for 15 min.

Raman spectroscopy is used to further assess film morphology and to study the electronic structure of P3HT in blends.^24,25^ The double bonds intrinsic to π-conjugation along a polymer backbone have high polarizable electron density along the bond axis, which causes a large Raman intensity in the vibrations associated with these double bonds. Furthermore, vibrational spectroscopy is highly sensitive to structural and conformational changes, where small changes in geometry of hundredths of angstroms are detectable.^26^ The P3HT:FNTz-T_eh_-FA blend Raman spectra are dominated by the intense P3HT B band at 1450 cm^–1^, and the bands related to the FNTz-T_eh_-FA are only weakly visible (Figures 3 and S4). The Raman spectra of linear polymer chains have been previously described using Zerbi’s Effective Conjugation Coordinate Model,^27,28^ where the symmetric C=C stretching mode along the conjugated backbone can be used as a direct probe of electronic structure, conjugation length, and conformation. The lack of change in the P3HT B band across the P3HT:FNTz-T_eh_-FA blends fabricated from different solvents indicates that the conjugation length of the P3HT chains remains the same irrespective of processing solvent, and this is consistent with the lack of shift observed in the steady-state absorption spectra from pristine P3HT to each blend.

Interestingly, the P3HT B band in all of the P3HT:FNTz-T_eh_-FA blends is broader (fwhm = 44–46 cm^–1^) than that of the control P3HT:PCBM blend (fwhm = 32 cm^–1^), with the distinct formation of a higher wavenumber shoulder at ∼1470 cm^–1^ (Figure 3). After decomposing the band into two, one centered at 1450 cm^–1^ and one at 1470 cm^–1^, we note that in each blend the area of the 1470 cm^–1^ band is proportional to the area of the 1285 cm^–1^ FNTz-T_eh_-FA band. This implies that the broadening of the P3HT B band is due to the overlap with an acceptor vibrational band, rather than because of disruption of the polymer packing. This consistency of the P3HT morphology is in agreement with the steady-state absorption band, showing little shift between the pristine P3HT and the FNTz-T_eh_-FA blends.

**Figure 4:**
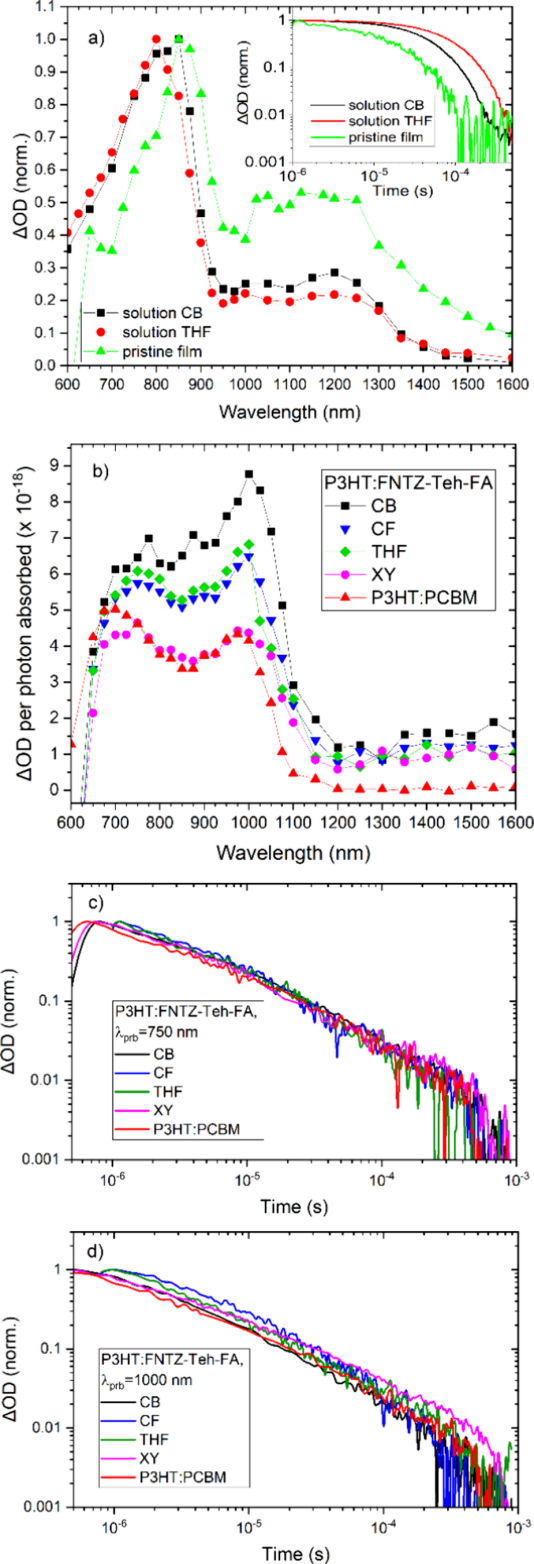
(a) The normalized TA spectra at 2 μs, measured for pristine FNTz-T_eh_-FA in solution and film. (b) The TA spectra at 2 μs corrected for number of photons absorbed, measured for P3HT:FNTz-T_eh_-FA blend films fabricated using different processing solvents, compared to a control P3HT:PCBM film. The normalized decay dynamics of the blend films probed at (c) 750 and (d) 1000 nm (the P3HT polaron) for the same sample. The excitation wavelength is 540 nm for films and 525 nm for solutions; the excitation density is 12–25 μJ cm^–2^. All blend films have a 1:1 weight ratio and are annealed at 135 °C for 15 min.

To assess the effects of the processing solvent on charge carrier populations and dynamics, we turn to transient absorption spectroscopy (TAS). TAS is used to directly monitor the optical absorption of photogenerated transient species, enabling the identity, yield, and decay mechanisms of these transient species to be ascertained. We focus here on μs-TAS, noting that the TA amplitude is directly proportional to the population of the photogenerated species. Indeed, it has been demonstrated that the amplitude of the polaron TA signal at 1 μs is proportional to both the short-circuit current and the external quantum efficiency of the corresponding photovoltaic device.^29,30^

Since triplet states are known to be prevalent in NFA blends,^31,32^ we first examine FNTz-T_eh_-FA in pristine solution and film, in order to ascertain its triplet characteristics (Figures 4a and S5). As expected, the triplet state is clearly visible in solution, with an intense TA band appearing at 800 nm, irrespective of solvent. Weaker features are present from 1000 to 1300 nm, but these possess the same kinetics as the TA maximum at 800 nm and thus belong to the same species. The monoexponential, oxygen-dependent decay kinetics enable a simple assignment of the 800 nm band to the FNTz-T_eh_-FA triplet state. The triplet lifetime does vary between solvents, from 47 μs in CB to 90 μs in THF, which may reflect some solvent-dependent pre-aggregation in solution.

The pristine FNTz-T_eh_-FA films also produce evidence of triplet states (Figures 4a and S5). The overall shape of the TA spectrum remains the same compared to solution, but the most intense band shifts to 860 nm in the pristine film (consistent with the red-shift from solution to film seen in the steady-state absorbance spectra), and the features from 1000–1300 nm become more prominent. Due to the fast second-order annihilation processes common in more condensed phases preceding the μs timescales we observe here, the triplet signal for the film is substantially weaker than that for the solution. Furthermore, the triplet lifetime is reduced to 20 μs.

The blend film TA spectra of P3HT:FNTz-T_eh_-FA, coated by using several different processing solvents, are shown in Figure 4b. The spectra are dominated by P3HT polaron features, as assessed by comparing to a control P3HT:PCBM TA spectrum. Indeed, the most intense P3HT polaron band at 1000 nm shows zero shifts as the processing solvent is altered.

Also present in the blend TA spectra is a feature at ∼760 nm. Both 1000 and 760 nm bands show power law decay dynamics (Figures 4c, 4d, and S6), suggesting bimolecular recombination of charge carriers.^33,34^ Bimodal polaron behavior has been observed in many polymer/acceptor blends.^35,36^ For P3HT:PCBM, three polaron bands have been identified depending upon the crystallinity of the film and the regioregularity of the polymer. Since we observe the 760 and 1000 nm polaron bands to have very similar kinetics (*vide infra*), we can discount the 760 nm band as being assigned to polarons in crystalline P3HT domains, previously observed at ∼700 nm.^36,37^ As such, the second polaron absorption at 760 nm is more akin to the 800 nm band previously observed by Guo et al.,^37^ and can therefore be assigned to polarons localized in mixed, disordered domains. In contrast, the 1000 nm band is attributed to polarons in relatively disordered (amorphous-like) P3HT domains, such as the interfacial area between crystalline P3HT domains and acceptor aggregates.^36,38^

The P3HT polaron decay dynamics are presented for the blends in different solvents in Figure 4c and 4d. Intriguingly, both P3HT polaron bands possess bimolecular recombination decays that are largely invariant as the processing solvent is changed. A power law gradient, α, of 0.69–0.71 is present for the P3HT:FNTz-T_eh_-FA 1000 nm polaron decay across the different solvents. The high α value suggests the presence of energetically shallow traps. However, the control P3HT:PCBM film also demonstrated a similar α, slightly larger than is typically observed for this blend.^34^ Furthermore, it should be noted that the decay kinetics of P3HT polarons are known to be highly sensitive to parameters such as polymer molecular weight, regioregularity, and processing conditions.^34^ The similarities between the kinetics of P3HT:FNTz-T_eh_-FA and P3HT:PCBM suggest that the recombination kinetics are largely dictated by the polymer, possibly because of its expected broader density of states influencing the charge carrier detrapping process.^38^ This is supported by the observation that the second polaron band at 760 nm has very similar kinetics to the 1000 nm polaron for each solvent (Figures 4c, 4d, and S6); both polarons observed are indeed related to interfacial domains.

Importantly, an assessment of the yield of 1000 nm P3HT polarons for each processing solvent reveals distinct differences. The P3HT:FNTz-T_eh_-FA blend fabricated from CB shows the highest P3HT polaron population at 2 μs, double that of the XY blend and P3HT:PCBM (Figure 4b). These results are consistent with measured photovoltaic device characteristics (Table S3, Figure S7), which show the XY blend to have the smallest short circuit current, *J*_SC_, and the CB blend to have the largest. Despite the doubling of the P3HT polaron population from the XY to CB blend, the short circuit current increases by 37%, suggesting that charge extraction effectively competes with bimolecular recombination.

The differences in charge yield with the different processing solvents cannot be explained by differences in bimolecular recombination rates, as these have been shown to be invariant with the processing solvent. Since we observe very little shift of the absorption onset with processing solvent, large changes in energy levels and thus driving force are not expected. As such, these differences in charge yield are likely related to differences in geminate recombination, which typically occurs prior to the time resolution of the μs-TAS. The implication that the XY blend has a greater degree of geminate recombination (and thus the fewest charges on μs time scales) is consistent with its higher PL quenching yield, which suggests small P3HT domains in which charge carriers cannot fully escape one another.

## Discussion

**Figure 5:**
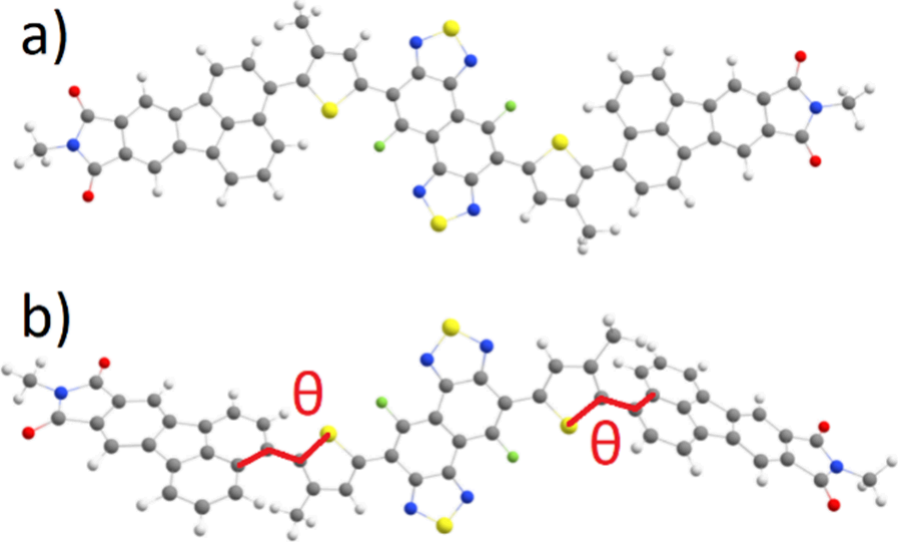
Calculated (a) planar and (b) global minimum conformations of FNTz-T_eh_-FA. In the global minimum conformation, there is a ±120° dihedral angle θ between the thiophene ring and the terminal unit.

The spectroscopy of FNTz-T_eh_-FA and its blends across several processing solvents has uncovered some interesting behavior: significant differences in charge carrier yields, for example, but invariant charge carrier dynamics. The experimental Raman spectra of the films are dominated by the P3HT component, and thus, to delve more specifically into the contributions from FNTz-T_eh_-FA, we turn to computational chemistry. Geometry optimizations and potential energy scan (Figures S8 and S9) calculations were thus performed for pristine FNTz-T_eh_-FA (B3LYP/6-31G(d)). Intriguingly, the energetically most favorable conformation of FNTz-T_eh_-FA is not planar but twisted due to steric effects, with a ±120° dihedral angle θ between the thiophene ring and the terminal unit (Figure 5). Typically, NFAs are designed to exhibit planar structures since enhanced conjugation allows for improved electron mobility.^39^ A common strategy to achieve planarity in non-fused-ring electron acceptors is by using non-covalent conformational locks.^40,41^ In FNTz-T_eh_-FA such a lock between the fluorine and sulfur atoms is present; however, FNTz-T_eh_-FA still does not achieve planarity. It should be noted that these calculations have been performed in-vacuo and packing effects in films could lead to a different, likely more planar conformation. However, many other NFAs—including both fused- and non-fused-ring NFAs—are calculated to be planar even in-vacuo.^42−48^

**Figure 6:**
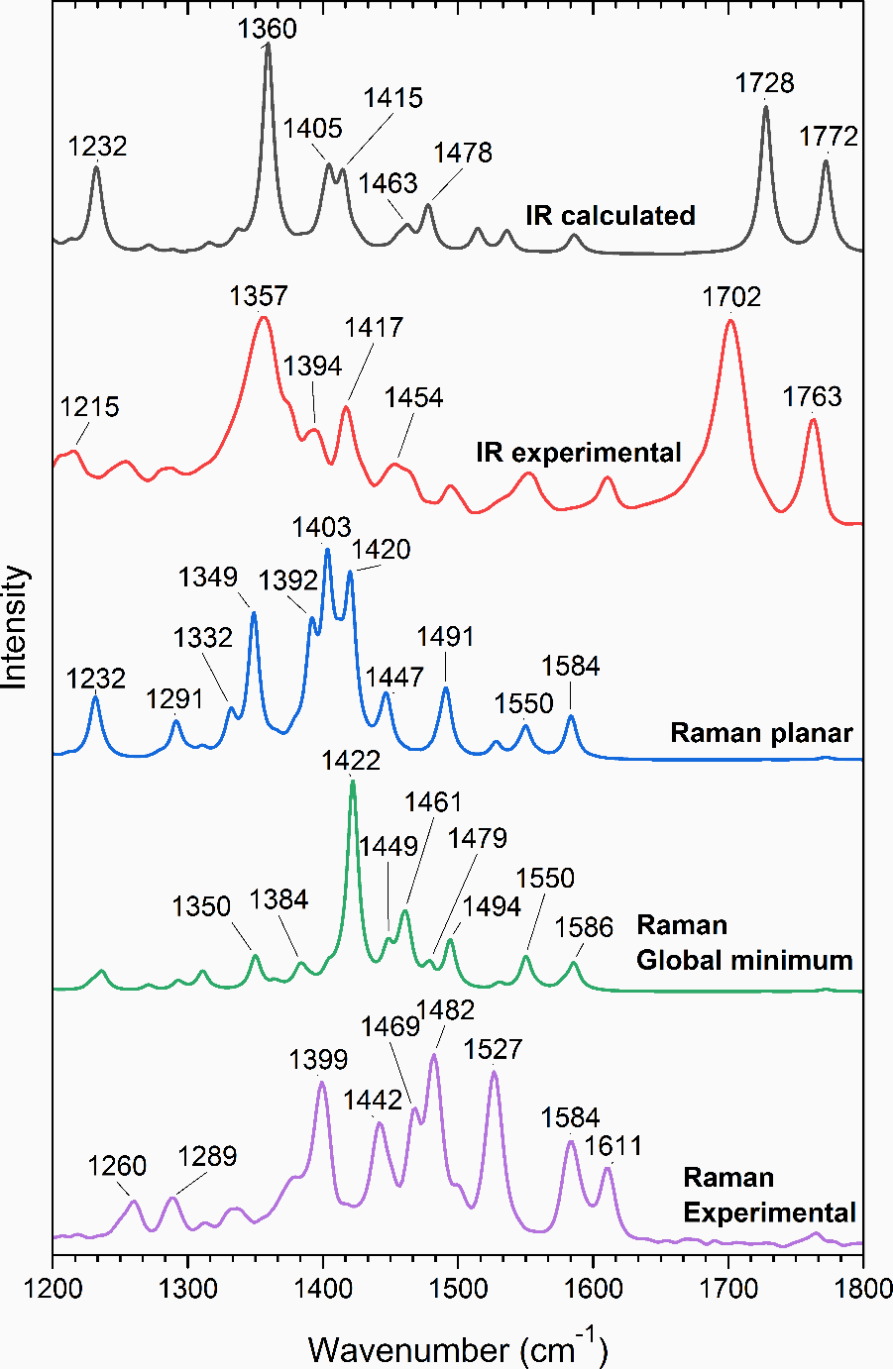
Calculated IR spectrum for the global minimum conformation and the experimentally measured IR spectrum of pristine FNTz-T_eh_-FA powder. Calculated Raman spectra for planar and global minimum conformations and the experimentally measured FT-Raman spectrum of the pristine FNTz-T_eh_-FA powder (λ_exc_ = 1064 nm). The measured spectrum is an amalgamation of the two calculated spectra based on the planar and global minimum conformations.

To test the validity of our calculated global minimum structure, we calculate the corresponding vibrational spectra and compare these to experimental results for pristine FNTz-T_eh_-FA in powder form. Calculations of the IR spectrum of FNTz-T_eh_-FA using the global minimum conformation produce a reasonably good match with experimental measurements of powder, with five of the six most intense bands matching within 20 cm^–1^ (Figure 6). The intensity pattern is also consistent between the calculated and experimental IR spectra. However, it is immediately obvious that the FT-Raman spectrum of pristine FNTz-T_eh_-FA does not match the global minimum structure. For example, one of the most prominent bands in the experimental spectrum at 1399 cm^–1^ has no clear corresponding band in the calculated global minimum Raman spectrum. Furthermore, the most intense band in the calculated spectrum at 1422 cm^–1^ (assigned to the symmetric C=C thiophene stretch) corresponds to the 1442 cm^–1^ band in the experimental spectrum but is only one-fifth in intensity. Unlike IR spectroscopy, Raman spectroscopy is highly sensitive to π systems and, hence, conjugation. The lack of correspondence between the calculated and experimental structures suggests that the calculation has not captured the correct backbone conformation.

To probe this observation further, FNTz-T_eh_-FA Raman spectra were calculated for a variety of different conformations, including local minima conformations (Figure S10) and a fully planar structure. Interestingly, none of the calculated spectra exhibited a good match with the experimental data. Instead, the experimental Raman spectra appear to be a combination of the global minimum and planar conformation calculated spectra. As previously mentioned, for example, the 1422 cm^–1^ band calculated from the global minimum conformation corresponds to the 1442 cm^–1^ band in the experimental spectrum and has been assigned to the symmetric C=C stretch localized on the thiophene rings. Meanwhile, the 1392 cm^–1^ band calculated from the planar conformation, assigned to the symmetric C=C stretch localized on the terminal rings, corresponds to the 1399 cm^–1^ band in the experimental data. The lack of correspondence to a single conformer strongly suggests that multiple conformations are present even in pristine FNTz-T_eh_-FA films. Furthermore, as donor:acceptor blends tend to have less crystallinity than pristine films due to the disruption of molecular packing with the presence of two components,^50^ blend films containing FNTz-T_eh_-FA are expected to have an even higher likelihood of multiple FNTz-T_eh_-FA conformations than the pristine films.

The similar TAS decay dynamics across the processing solvents may also be correlated with FNTz-T_eh_-FA’s non-planarity. The predicted NFA non-planarity seems to occur irrespective of processing solvent, and a range of aggregate sizes are observed: thus we do not achieve a more optimal morphology using one solvent over another. Since all blends have this dispersive environment with multiple NFA conformations, while still maintaining the intrinsic crystallinity of P3HT, the bimolecular recombination we observe is largely invariant.

The presence of multiple conformations of FNTz-T_eh_-FA in films has some important implications for the field of organic photovoltaics. Sensitivity to crystallinity and morphology can lead to strongly varying device characteristics. In contrast, the non-planar NFA FNTz-T_eh_-FA is largely insensitive to processing solvent, with only subtle changes in morphology and charge carrier bimolecular recombination. As such, it is possible that non-planar NFAs could present a promising strategy for upscaling of organic photovoltaics. Such acceptors would circumvent the issue of planarity–solubility trade-off, simultaneously offering good solubility and good charge carrier transport.^51^

## Conclusions

In summary, we demonstrate a non-fullerene acceptor specifically designed to match with polymer donor P3HT for green-absorbing agrivoltaics. P3HT:FNTz-T_eh_-FA creates a range of aggregate sizes across a variety of organic processing solvents while still enabling the polymer to maintain its relatively crystalline morphology. This facilitates similar P3HT polaron decay dynamics regardless of the processing solvent used. From a manufacturing perspective, this insensitivity of the charge carrier decay dynamics to processing solvent is very valuable. Computational modeling and Raman spectroscopy reveal that FNTz-T_eh_-FA is non-planar and exhibits multiple conformations in films. This large conformational dispersity means that an averaged charge carrier decay is observed, and this helps to explain the high tolerance to morphology variation.

## Supporting Information Available

Synthesis procedure, synthetic complexity analysis, steady-state absorbance data, AFM data, Raman data, triplet-state characterization, TA decay dynamics comparison, device characterization, and computational chemistry results (PDF)
