# Supplementary material for: Invariant Charge Carrier Dynamics Using a Non-Planar Non-Fullerene Acceptor across Multiple Processing Solvents
Source: J Phys Chem C Nanomater Interfaces. 2024 Apr 11;128(16):6758–66. doi: 10.1021/acs.jpcc.4c00708 (PMC11056975; doi:10.1021/acs.jpcc.4c00708)
Supplement: Supplementary file 1 — jp4c00708_si_001.pdf [file jp4c00708_si_001.pdf]

SUPPORTING INFORMATION

Invariant Charge Carrier Dynamics Using a Non-Planar Non-Fullerene Acceptor across Multiple Processing Solvents

Hristo Ivov Gonev,[a] Elena Jones,[a] Chia-Yu Chang,[a] Yutaka Ie,[b] Shreyam Chatterjee,[b,] Tracey M. Clarke[a,]**

[a] Affiliation 1: Department of Chemistry, University College London, Christopher Ingold Building, London, WC1H 0AJ, United Kingdom

[b] Affiliation 2: The Institute of Scientific and Industrial Research (SANKEN), Osaka University, 8-1 Mihogaoka, Ibaraki, Osaka 567-0047, Japan

*Corresponding authors: Tracey M. Clarke (tracey.clarke@ucl.ac.uk) and Shreyam Chatterjee (shreyam@sanken.osaka-u.ac.jp)

Table S1. Synthetic complexity of FNTz-T_{eh}-FA's building units, where NSS is the number of synthetic steps, RY are the reciprocal yields, NUO is the number of unit operations required for isolation/purification, NCC is the number of column chromatographic purifications required and NHC is the number of hazardous chemicals used. Values used for the normalization: NSS_{max} = 13; RY_{max} = 77; NUO_{max} = 25; NCC_{max} = 9; NHC_{max} = 36.

Building units	Absolute values					Normalised Values				
	NSS	RY	NUO	NCC	NHC	NSS	RY	NUO	NCC	NHC
**FNTz-Br**	11	1.54	15	3	11	0.84	0.1	0.6	0.33	0.30
**FA-B**	5	1.25	13	3	7	0.38	0.96	0.52	0.33	0.19

Table S2. Synthetic complexity of FNTz-T_{eh}-FA compared to other NFAs, where NSS is the number of synthetic steps, RY are the reciprocal yields, NUO is the number of unit operations required for isolation/purification, NCC is the number of column chromatographic purifications required and NHC is the number of hazardous chemicals used. Values used for the normalization: NSS_{max} = 17; RY_{max} = 25; NUO_{max} = 29; NCC_{max} = 7; NHC_{max} = 31.

**Target Compound**	**Absolute values**					**Normalized Values**					**SC**
	**NSS**	**RY**	**NUO**	**NCC**	**NHC**	**NSS**	**RY**	**NUO**	**NCC**	**NHC**	
**FNTz-T_{eh}-FA**	17	2.8	28	6	18	0.8	0.32	0.97	0.86	0.58	**76.3**
**O-IDTBR**	17	6.7	28	7	28	1	0.59	0.97	1	0.93	**88.6**
**Y6**	17	25	29	6	30	1	1	1	0.86	1	**97.9**
**ITIC**	10	4	22	6	17	0.59	0.43	0.76	0.86	0.57	**61.3**

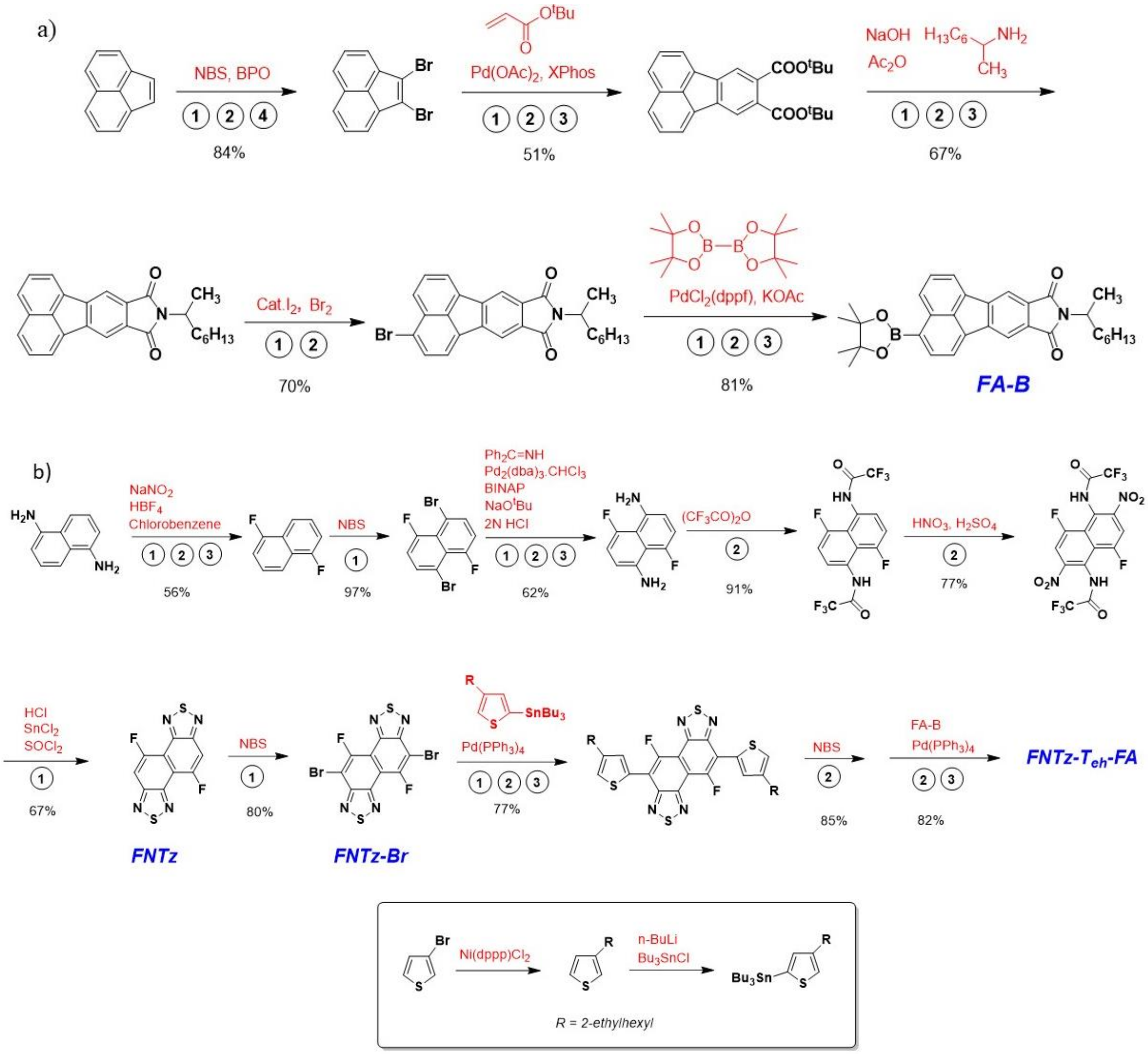

Figure S1. Synthesis of FA-B (a) and FNTz-T_{eh}-FA (b). 1 signifies quenching/neutralisation, 2 – extraction, 3 – column chromatography, 4 – recrystallization, 5 – distillation/sublimation.

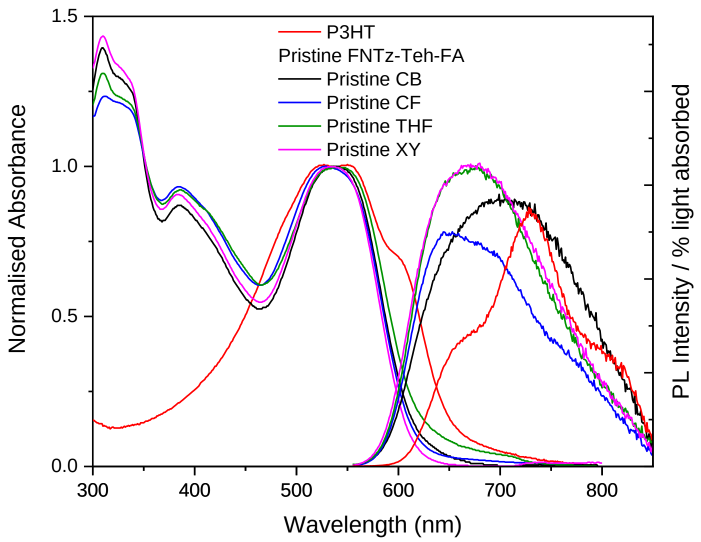

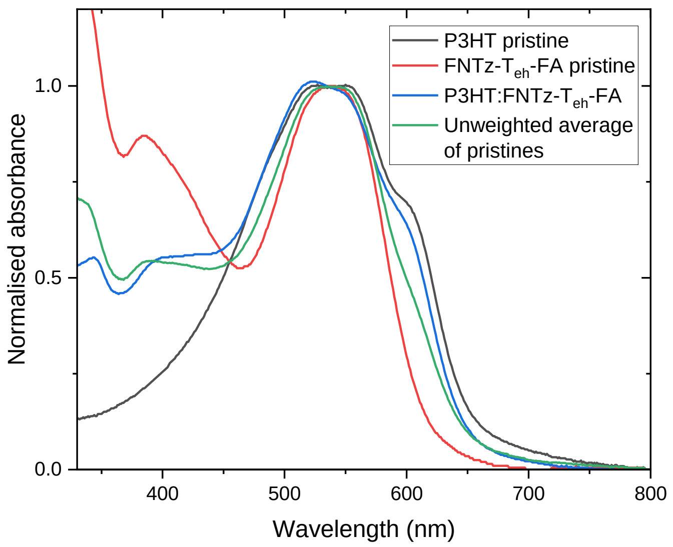

Figure S2. Above - the steady-state absorption and photoluminescence spectra of FNTz-T$_{eh}$-FA films fabricated using different processing solvents. The PL spectra were measured using an excitation wavelength of 540 nm and are corrected for the percentage of light absorbed at that wavelength. Also included is the pristine P3HT control sample. Below – the unweighted average of the absorbance spectra of pristine P3HT and FNTz-T$_{eh}$-FA, showing close similarity to the blend film in terms of relative intensity of the FNTz-T$_{eh}$-FA features.

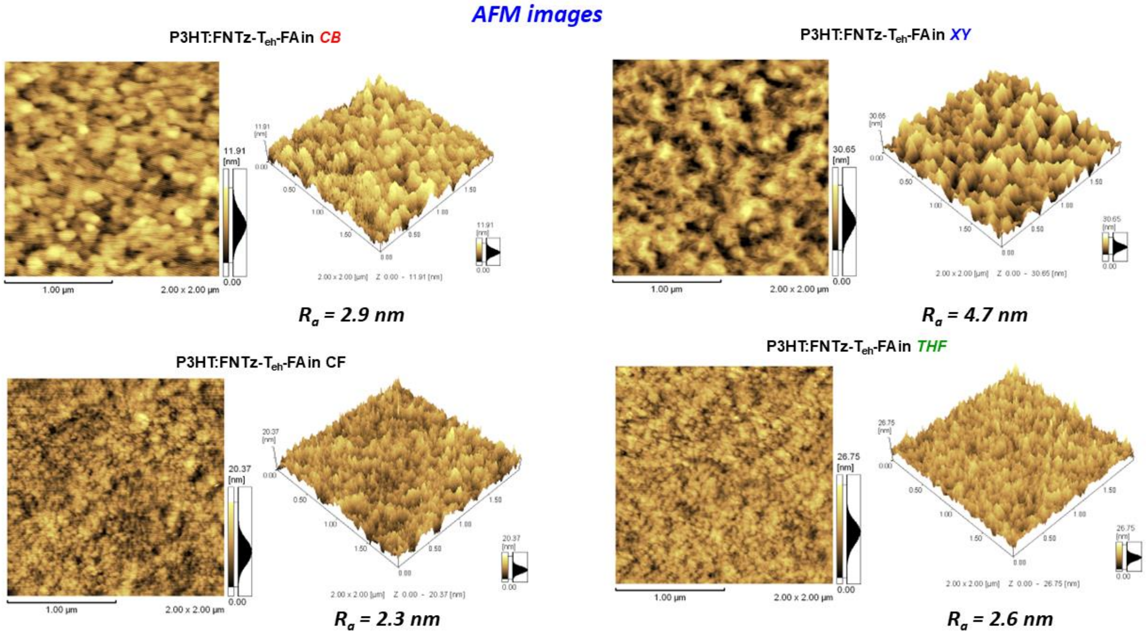

Figure S3. Atomic force microscopy images for the P3HT:FNTz-T_{eh}-FA blend films prepared in different processing solvents. R_a values for each film are indicated.

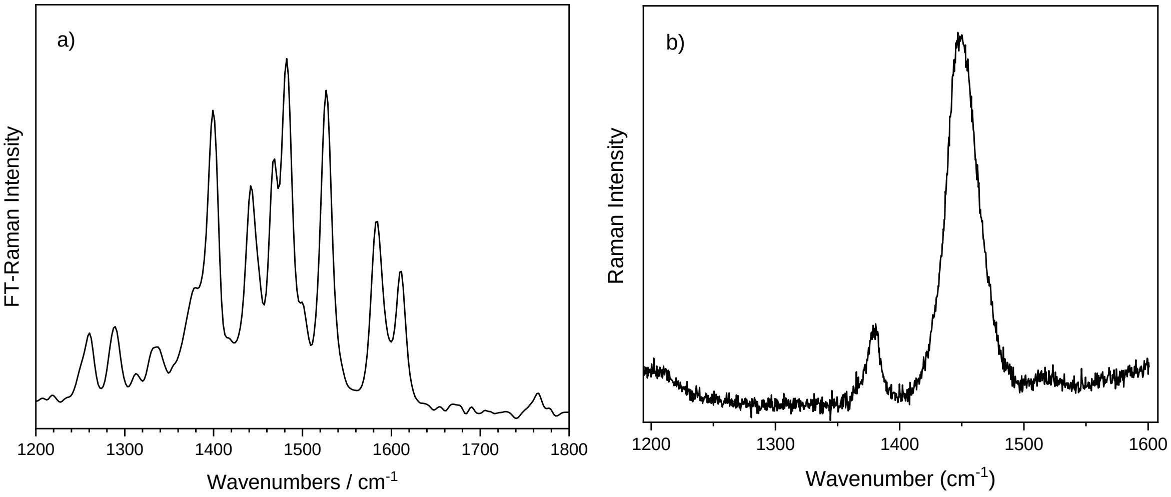

Figure S4. The FT-Raman spectrum of pristine FNTz-T_{eh}-FA powder (a) (λ_{exc} = 1064 nm) and the Raman spectrum of pristine P3HT (b) (λ_{exc} = 532 nm).

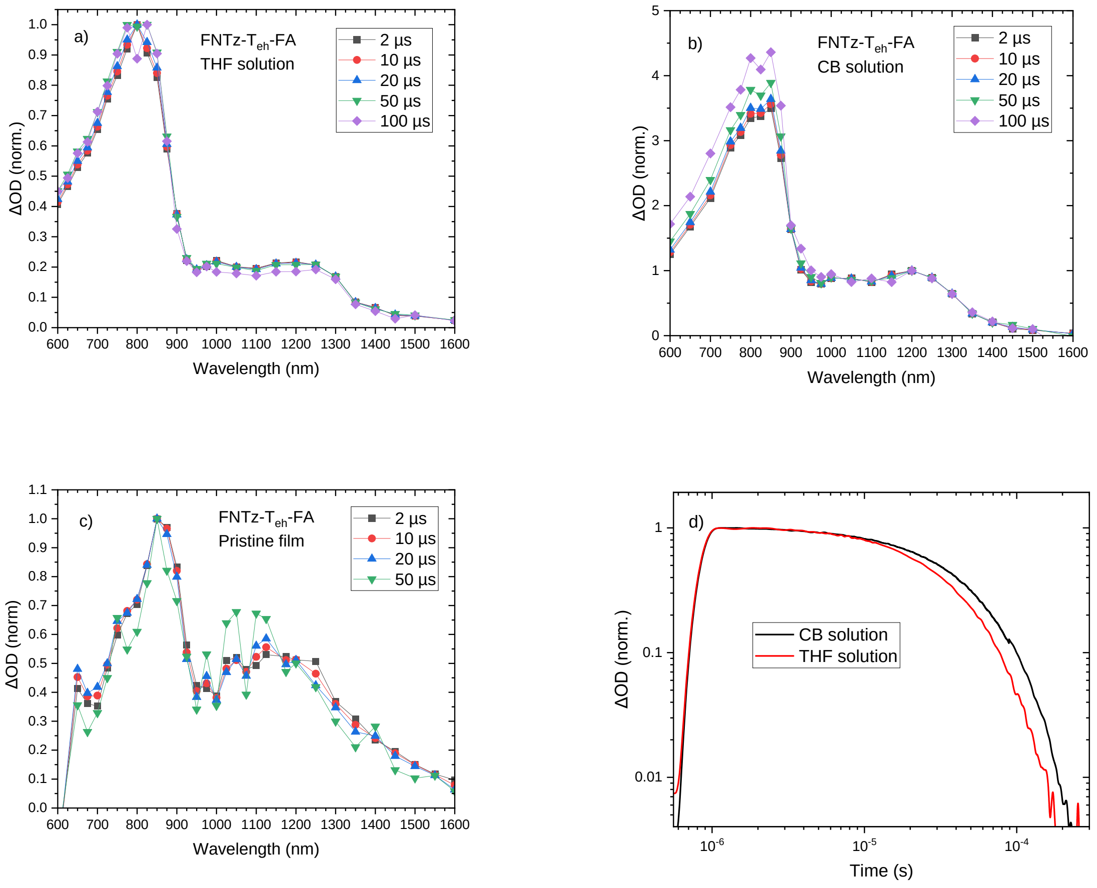

Figure S5. The normalised TA spectra of FNTz-T$_{eh}$-FA in two representative solvents, THF (a) and CB (b) as well as the pristine film (c), all three possessing only triplet signals. Also shown are the classical monoexponential decay kinetics of the triplets in solution (d). The excitation wavelength was 540 nm and the excitation density was 12 μJ cm^{-2}.

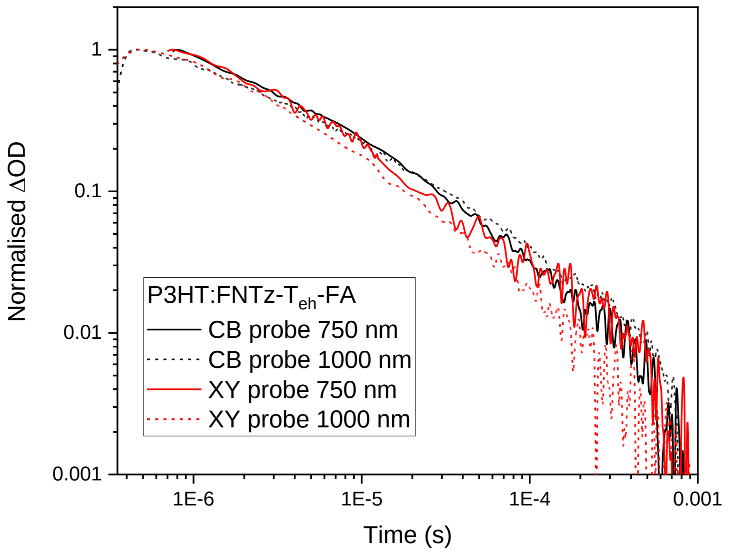

Figure S6. A comparison between the 750 nm and 1000 nm P3HT polaron kinetics for blend films fabricated with two representative solvents. An excitation wavelength of 540 nm was used and an excitation density of ~20 μJ cm^{-2}.

Table S3. Device parameters for P3HT:FNTz-T_{eh}-FA fabricated with different processing solvents. The standard deviations are indicated within the bracket from six devices fabricated with each solvent condition.

**Processing solvent**	**PCE (%)**	**J_{SC} (mA cm^{-2})**	**V_{OC} (V)**	**FF**
**CB**	3.11 ± 0.08	6.15 ± 0.09	0.92 ± 0.01	0.55 ± 0.01
**CF**	2.45 ± 0.02	5.07 ± 0.04	0.92 ± 0.01	0.53 ± 0.02
**THF**	2.71 ± 0.03	5.71 ± 0.06	0.89 ± 0.02	0.53 ± 0.01
**XY**	2.43 ± 0.05	4.50 ± 0.07	0.90 ± 0.01	0.60 ± 0.01

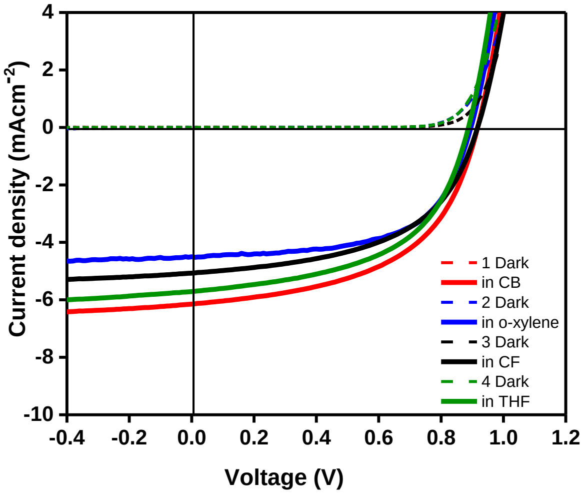

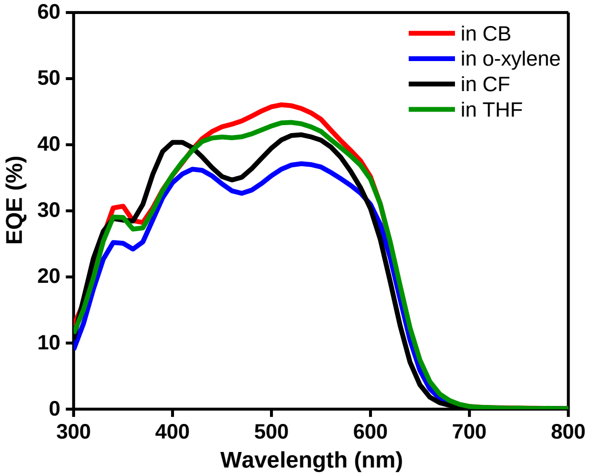

Figure S7. The JV curves (left) and EQE (right) for the P3HT:FNTz-T$_{eh}$-FA devices prepared in different processing solvents.

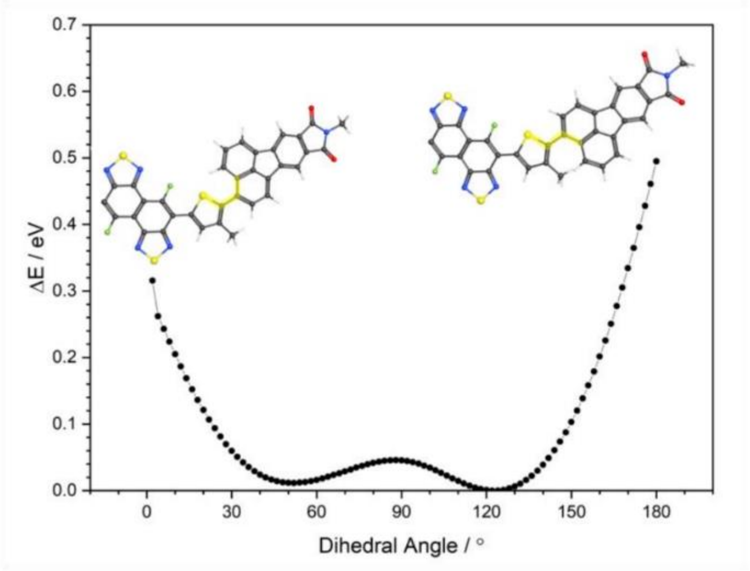

Figure S8. Potential energy scan of dihedral angle α with a zoom-in of the starting and final structures. The rest of the molecule is not shown as its geometry remains fixed. Energies have been plotted relative to the minimum energy point.

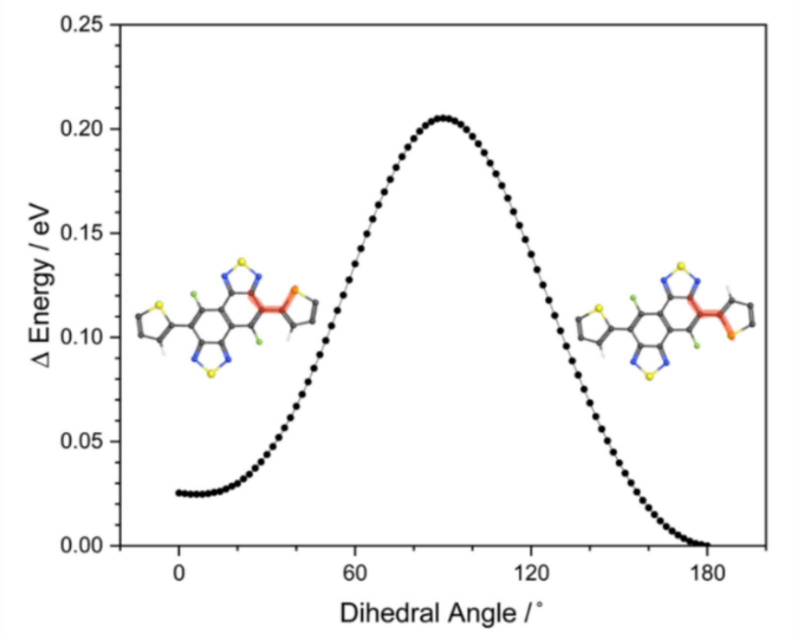

Figure S9. Potential energy scan of the dihedral angle shown in red. A zoom-in on the FNTz central unit is included to illustrate the thiophene ring changing from the sulphur atom being trans to the fluorine atom at 0°, to being cis to it at 180°. Energy is plotted relative to the minimum energy point on graph. The lower potential energy at the cis orientation is due to the presence of a conformational lock between the S and F atoms.

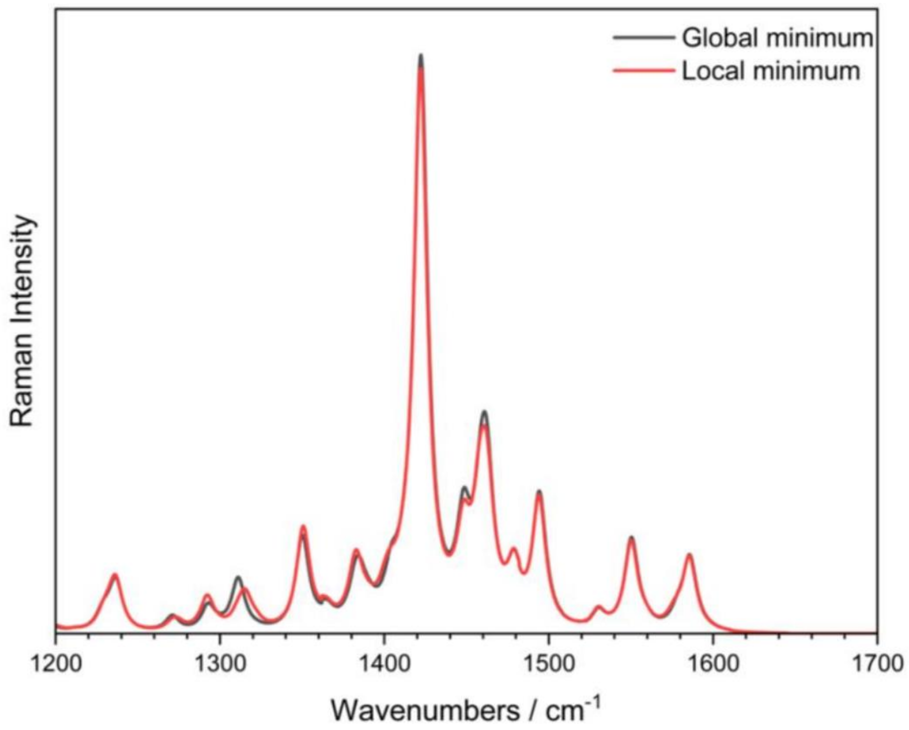

Figure S10. Calculated Raman spectra for the global and local minimum conformations of FNTz-T_{eh}-FA, as found in figure S8.
